# Efficacy and Safety of Aronia, Red Ginseng, Shiitake Mushroom, and Nattokinase Mixture on Insulin Resistance in Prediabetic Adults: A Randomized, Double-Blinded, Placebo-Controlled Trial

**DOI:** 10.3390/foods10071558

**Published:** 2021-07-05

**Authors:** Sunmin Park, Chan-Joong Kim, Ki-Chan Ha, Hyang-Im Baek, Hye-Jeong Yang, Min-Jung Kim, Soo-Jung Park

**Affiliations:** 1 Department of Food & Nutrition, Obesity/Diabetes Center, Hoseo University, Asan 31499, Korea; 2 Urban Agriculture Research Division, National Institute of Horticultural and Herbal Science, Wanju 55365, Korea; chan7653@naver.com; 3 Healthcare Claims & Management Incorporation, Jeonju 54858, Korea; omphalos9121@hanmail.net (K.-C.H.); hyangim100@gmail.com (H.-I.B.); 4 Food Functional Research Division, Korean Food Research Institutes, Wanju 55365, Korea; yhj@kfri.re.kr (H.-J.Y.); kmj@kfri.re.kr (M.-J.K.); 5 Department of Sasang Constitutional Medicine, Woosuk University Korean Medicine Hospital, Jeonju 55338, Korea

**Keywords:** *Aronia melanocarpa* berries, red ginseng, insulin sensitivity, liver damage, inflammation, type 2 diabetes

## Abstract

We determined whether oral consumption of Aronia, red ginseng, shiitake mushroom, and nattokinase mixture (3.4: 4.1: 2.4: 0.1 *w*/*w*; AGM) improved glucose metabolism and insulin resistance in prediabetic adults in a 12-week randomized, double-blinded clinical trial. Participants with fasting serum glucose concentrations of 100–140 mg/dL were recruited and randomly assigned to an AGM or placebo group. Participants of the AGM group (*n* = 40) were given an AGM granule containing 4 g of freeze-dried Aronia, red ginseng, shiitake mushroom, and nattokinase (3.4: 4.1: 2.4: 0.1 *w*/*w*) twice daily for 12 weeks, and the placebo group participants (*n* = 40) were provided with corn starch granules identical in appearance, weight, and flavor for 12 weeks. Serum glucose and insulin concentrations were measured during oral-glucose tolerance tests (OGTT) after administering 75 g of glucose in a fasted state. HOMA-IR, liver damage, and inflammation indices were determined, and safety parameters and adverse reactions were assessed. As determined by OGTT, serum glucose concentrations were not significantly different between the AGM and placebo groups after the intervention. However, changes in serum insulin concentrations in the fasted state and Homeostatic model assessment-insulin resistance (HOMA-IR) index after the intervention were significantly lower in the AGM group than in the placebo group (−3.07 ± 7.06 vs. 0.05 ± 6.12, *p* = 0.043 for serum insulin; −0.85 ± 2.14 vs. 0.07 ± 1.92, *p* = 0.049 for HOMA-IR). Serum adiponectin concentrations were reduced by intervention in the placebo group but not in the AGM group. Changes in liver damage indexes, including serum activities of the γ-glutamyl transferase, alanine aminotransferase, and aspartate aminotransferase, were lower in the AGM group and significantly reduced in the AGM group more than in the placebo group (*p* < 0.05). Changes in serum high sensitive-C-reactive protein concentrations in AGM and placebo groups were significantly different (−0.12 ± 0.81 vs. 0.51 ± 1.95, *p* = 0.06). In conclusion, AGM possibly improves insulin sensitivity and β-cell function and reduces liver damage and inflammation in prediabetic adults.

## 1. Introduction

The prevalence of type 2 diabetes is increasing worldwide and is more remarkable in Asians than in Caucasians. In Korean adults, its prevalence increased from 4.5% to 13.8% in the last 60 years, while it doubled over the same period in those aged >65 years [1]. Moreover, the percentage of Korean adults with impaired glucose intolerance was 26.9% in 2018 [1]. Although obesity is positively associated with the prevalence of type 2 diabetes, in 2010, the mean body mass index (BMI) of type 2 diabetes patients was 25.2 kg/m^2^ in Korea [1]. The higher prevalence of type 2 diabetes in Asians is related to less insulin secretion and β–cell mass than Caucasians. Therefore, in Asians, serum glucose concentrations quickly increase when insulin resistance increases due to aging, obesity, estrogen deficiency, inflammation, oxidative stress, or a sedentary lifestyle. Of these factors, aging and menopause in women inevitably increase insulin resistance and interact with environmental factors to exacerbate inflammation and oxidative stress. As a result, the elderly are susceptible to glucose metabolism impairment.

Adults with type 2 diabetes usually have one or more of the following comorbidities, that is, obesity, hypertension, dyslipidemia, or liver disease (hepatic steatosis). Of type 2 diabetic patients, 28.8% are obese and have hypertension, whereas 26.0% are obese and have dyslipidemia [1]. Obesity is generally considered the primary trigger of insulin resistance, but it is unclear whether reducing insulin resistance protects against obesity, although it positively affects type 2 diabetes, hypertension, dyslipidemia, and liver diseases. In addition to insulin resistance, type 2 diabetes is associated with reduced glucose-stimulated insulin secretion and β-cell mass, and reduced insulin resistance compensates for low insulin secretion, promotes β-cell function, and prevents β-cell exhaustion. Therefore, reductions in insulin resistance play a crucial role in preventing glucose intolerance to type 2 diabetes.

*Aronia melanocarpa* (black chokeberry) berries, red ginseng, and *Lentinula edodes* (Shiitake mushrooms) contain anthocyanins, ginsenosides, saponins, β-glucans, vitamin D, and other phytochemicals, and animal studies have shown they all have anti-inflammatory and anti-oxidative activities [2]. Furthermore, animal and clinical studies have shown *that Aronia* and red ginseng extracts reduce fat deposits, improve insulin resistance, and promote glucose and lipid metabolism [2,3,4,5,6]. β-Glucans and vitamin D in mushrooms enhance glucose metabolism by promoting insulin sensitivity, insulin secretion, and immunity via rodents’ intestinal microbiome [7,8,9]. Asians have low serum concentrations of 25-hydroxycholecalciferol (an indicator of vitamin D status) due to insufficient sun exposure [10]. Ultraviolet-irradiated mushrooms are an excellent source of vitamin D, but their bioavailability for vitamin D remains unclear. It was suggested in a recent review that vitamin D2 (ergocholecalciferol) in ultraviolet-irradiated Shiitake mushrooms has lower bioavailability than vitamin D3, although ergocholecalciferol is absorbed in the small intestine and converted into 25-hydroxy cholecalciferol in the liver [11]. On the other hand, nattokinase intake dissolves blood clots and reduces blood pressure in a randomized, double-blind, placebo-controlled clinical trial [12].

Oral intake of an *Aronia melanocarpa* berry, red ginseng, Shiitake mushroom, and nattokinase mixture has been reported to improve glucose metabolism by reducing insulin resistance and potentiating insulin secretion and also to enhance lipid metabolism and inflammation in insulin-deficient type 2 diabetic rats. These improvements were ascribed to changes in gut microbiome composition [13]. We hypothesized that the consumption of Aronia, red ginseng, Shiitake mushroom, and nattokinase mixture might improve glucose metabolism and diminish insulin resistance in prediabetic adults. This hypothesis was examined using a 12-week randomized, double-blinded clinical trial. The present study aimed to investigate the efficacy of the mixture on glucose metabolism, liver damage, and inflammatory status after a 12-week intervention.

## 2. Materials and Methods

### 2.1. Sample Preparation

Aronia, red ginseng, and Shiitake mushrooms were washed, dried at room temperature, freeze-dried, and powdered. Aronia, red ginseng, and Shiitake mushroom powder mixture (AGM) was prepared by mixing powdered red ginseng, Aronia, shitake mushrooms, and nattokinase (4.1: 3.4: 2.4:0.1, *w*/*w*; Chakreis, YD Nutraceuticals Ltd., Yongin-si, Korea) to contain 4 g fine granules per pack. This process was performed by YD Nutraceuticals Ltd. (Yongin-si, Korea). The nattokinase had 850 fibrin degradation units/g. The AGM contained ~2.0 g sugar, 0.5 g protein, 1.6 g dietary fiber, 0.25 g water, and other components totaling 0.35 g in one 4 g serving and had a calorific content of 10 kcal/4 g.

A placebo pack was made using colored and flavored granules of 4 g corn starch to match the AGM granules for weight, color, and flavor. A preliminary study showed 8 g of corn starch did not elevate serum glucose concentrations at 30, 60, or 120 min as determined by oral glucose tolerance testing (OGTT).

### 2.2. Measurement of Indicative Compounds in AGM

The total anthocyanin contents in AGM were measured using a pH differential method as previously described [14]. The AGM index compounds were cyanidin 3-O-galactoside, cyanidin 3-O-glucose, and cyanidin 3-O-arabinose for Aronia and ginsenosides Rb1 and Rb3 for red ginseng. Cyanidin 3-O-galactoside, cyanidin 3-O-glucose, and cyanidin 3-O-arabinose contents were measured by high performance liquid chromatography (HPLC) (Agilent Technologies, Santa Clara, CA, USA) using a Luna C18 column (4.6 × 250 mm, 5 µm; Phenomenex, Torrance, CA, USA) [14]. The mobile phase consisted of distilled water (A) and acetonitrile (B) mixes, and elution was performed using the following program: 0 min, A:B 100:0 (*v*/*v*); 10 min, A:B 88:12; 20 min, A:B 80:20; 35 min, A:B 60:40; 40 min, A:B 10:90; and 42 min, A:B 0:100. The mobile phase flow rate was 1.0 mL/min, the column temperature was 30 °C, injection volume was 10 μL, and UV detection was performed at 254 nm. Ginsenoside Rb1 and Rg3 levels were also measured by HPLC using the same conditions but at a flow rate of 0.6 mL using a UV detector at 203 nm [15]. Standards are cyanidin 3-O-galactoside, cyanidin 3-O-glucose, cyanidin 3-O-arabinose, and ginsenoside Rb1 and Rg3 were purchased from Sigma-Aldrich Co. (St. Louise, MO, USA). β-Glucan contents were determined using a b-glucan assay kit (Megazyme, Sydney, Australia) using an enzymatic method, and vitamin D2 contents were determined by HPLC [16] at KOTITI Testing & Research Institute (Sungnam, Korea). AGM contained 21.6 mg anthocyanins, 6.22 mg cyanidin 3-O-galactoside, 0.33 mg cyanidin 3-O-glucose, 1.53 mg cyanidin 3-O-arabinose, 4.97 mg ginsenoside Rb1, 2.5 mg ginsenoside Rg3 per g powder, 238 mg β-glucan, and 4.05 μg vitamin D2.

### 2.3. Participants

Participants were recruited at Woo Suk University Korean Medicine Hospital (Jeonju, Korea) through local advertisements. All provided written, informed consent and underwent initial screening to ensure they met the study inclusion criteria: age 19–70 years, fasting serum glucose concentration 100–140 mg/dL, and a body mass index of 18.5–35 kg/m^2^. The exclusion criteria were ≥6.5% hemoglobin A1c (HbA1c), alanine aminotransferase (ALT) 100 IU and aspartate aminotransferase (AST) 100 IU, lactating women, a possible pregnant status, alcohol or drug abuse, a diagnosis of type 2 diabetes, or receipt of ginseng or another dietary supplement known to have a hypoglycemic or potential hypoglycemic effect, or receipt of any anti-obesity agent, blood pressure, or lipid-lowering agent. Those that had participated in another clinical trial during the 3 months before screening were also excluded.

### 2.4. Study Design

Participants were enrolled from 4 November 2019, and enrollment was completed on 28 May 2020. The study was designed as a randomized, double-blind, placebo-controlled clinical trial. General information was recorded at screening, and participants were randomly divided into two groups and randomly allocated to an AGM group (*n* = 40) or a placebo group (*n* = 40) using computer-generated random numbers. Participants, investigators, and nurses were blinded to AGM or placebo throughout the intervention. According to the instruction, participants took a pack twice daily for 12 weeks and visited the clinic at 12 weeks to assess the efficacy and safety of the intervention. The leftover supplements were returned to the nurses to determine the compliance check for the intervention. Participants were encouraged to maintain their lifestyles, physical activity levels, and diets throughout the study period. They were not allowed to take any dietary supplements, including functional foods, vitamins, and minerals.

### 2.5. Sample Size Calculation

Previous studies have reported fasting serum glucose concentrations fell by 8.58 mg/dL among the prediabetic participants that took 8 g AGM granules per day and by 2.9 mg/dL for placebo controls (standard deviations were 7.5 mg in both groups). The sample size of the present study was estimated using the following parameters: level of significance, α = 0.05, β = 0.2, and power =80% for participants in the AGM and placebo groups, λ = 1. The calculated sample size was 28 per group, and thus, assuming a dropout rate of 30%, 40 participants were recruited per group.

### 2.6. Intervention and Primary and Secondary Outcomes

Based on a previous animal study [13], two packs (4 g/each) of AGM or placebo were administered daily for 12 weeks. Participants met our research team on four occasions: during screening (week −3), at treatment baseline for randomization (week 0), midway through treatment (week 6), and after 12 weeks of treatment (week 12). Compliances with interventions were calculated by dividing the number of packs consumed by the number prescribed.

Primary outcomes were measured in serum glucose concentrations at 0, 30, 60, 90, and 120 min after an oral intake of 75 g glucose at 0- and 12-week. The changes in serum glucose concentrations were calculated at 0, 30, 60, 90, and 120 min. Secondary outcomes were changes in serum insulin concentrations at 0, 30, and 60 min during OGTT, changes in the area under the curve (AUC) of serum glucose and insulin concentrations, and changes in other glucose-related biomarkers, e.g., serum C-peptide, insulin, and HbA1c concentrations at 12 weeks from the baseline. Homeostatic model assessment-insulin resistance (HOMA-IR) was also calculated by serum glucose and insulin concentrations at the fasting state.

### 2.7. Anthropometric Parameters, Blood Pressure Measurements, and Blood Collection

Body weights and heights were measured with participants unclothed. BMIs were calculated by dividing body weight by height squared (kg/m^2^). Blood pressures were measured on the left arms after a 10 min rest with patients seated, using an automatic blood pressure monitor.

### 2.8. OGTT and Biochemical Assays

After at least an 8 h fast, participants ingested a 75-g glucose solution. Venous blood specimens were collected 0, 30, 60, 90, and 120 min later, and serum glucose concentrations were measured using the glucose oxidase method using a Beckman Glucose Analyzer (Beckman Instruments). Serum insulin was determined by radioimmunoassay using a commercial kit from the ImmunoNuclear Corporation (Stillwater, MN, USA). AUC was calculated for serum glucose and insulin concentrations.

Serum C-peptide concentrations were measured using a two-site sandwich immunoassay using an ADVIA Centaur XP Immunoassay System (Siemens, Chicago, IL, USA). Hemoglobin (HbA1c) testing was performed using an immunoturbidimetric analyzer and a turbidimeter. Insulin resistance was calculated using the HOMA (homeostasis model assessment) of insulin resistance (HOMA-IR) method and the following equation: [Fasting insulin (μIU/mL) × Fasting glucose (mmol/L)/22.5)]. HOMA of β-cell function (HOMA-B) was calculated with the equation [20 × fasting serum insulin concentration (μIU/mL)/(fasting serum glucose concentrations (mmol/L) −3.5]. Oral deposition index was estimated by the equation [insulinogenic index/HOMA-IR] when the insulinogenic index was defined as the equation: [(serum insulin concentration at 30 min—serum insulin concentrations at 0 min)/( serum glucose concentration at 30 min—serum glucose concentrations at 0 min) during OGTT] [17]. The Matsuda index was calculated from 0- and 120-min data during OGTT, and its equation was [10,000/(fasting serum glucose × fasting serum insulin × mean serum glucose during OGTT × mean serum insulin during OGTT)^1/2^] [18]. Serum concentrations of free fatty acids (FFAs) were measured using an enzymatic assay based on the acetyl-CoA synthetase-acyl-CoA oxidase method and a Hitachi 7600 Autoanalyzer (Hitachi Ltd., Tokyo, Japan).

Liver functions were assessed using levels of gamma-glutamyl transferase (γ-GT), ALT, and AST, which were determined using a Hitachi 7600 Autoanalyzer (Hitachi Ltd., Tokyo), and inflammatory statuses were assessed using white blood cell (WBC) count and serum high-sensitive C-reactive peptide (hs-CRP) concentrations.

### 2.9. Dietary Intervention and Assessments of Dietary Intakes and Physical Activity Levels

Dietary intakes were estimated using 3-day food records (2 weekdays and 1 weekend day) for previous weeks at screening and after intervention for 6 and 12 weeks. Dietary energy values and nutrient contents from 3-day food records were calculated using the Computer-Aided Nutritional analysis program (CAN-pro 3.0, Korean Nutrition Society, Seoul). Physical activities were estimated using the Global Physical Activity Questionnaire at screening and on weeks 6 and 12.

### 2.10. Safety Assessment

Safety assessments were performed using electrocardiograms, vital signs (blood pressure and pulse rate), and biochemical assay results (total protein, alkaline phosphatase (ALP), hemoglobin level, hematocrit, platelet, blood urea nitrogen (BUN), and creatinine). Adverse events were documented and monitored throughout the trial.

### 2.11. Statistical Analysis

The statistical analysis was performed with SAS for Windows version 6.9.4 (SAS Institute, Cary, NC). Results are presented as numbers and percentages for categorical variables while means ± standard deviations (SD) for continuous variables. A full analysis set (FAS) was used for statistical analysis. The changes from baseline to 12 weeks were compared using paired t-tests in each AGM and placebo group. The changed values from baseline to 12-week were analyzed between the AGM and placebo groups using an independent t-test. The analysis of covariance (ANCOVA) test was used to assess the changed values from baseline to 12 weeks between the AGM and placebo groups after adjusting with body weight at baseline. Frequencies of adverse events in the two treatment groups were evaluated using a chi-square test. Statistical significance was accepted for *p* values < 0.05.

## 3. Results

### 3.1. Study Flow

**Figure 1:**
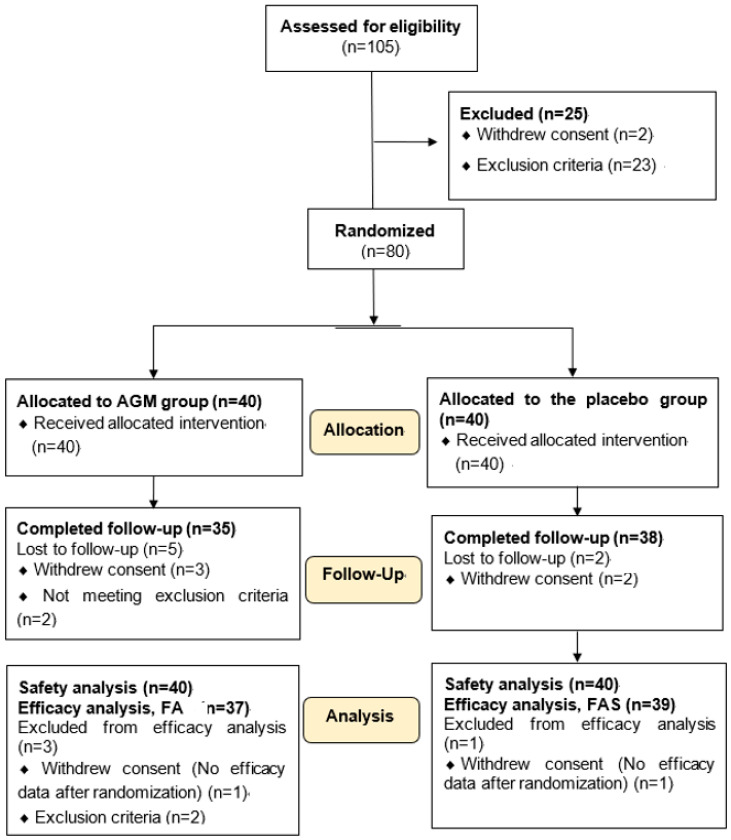
Flow diagram of participants in the process for the intervention. AGM, Mixture of Aronia, red ginseng, Shiitake mushroom, and nattokinase; FAS, the full analysis set.

After screening 105 volunteers, 25 were excluded due to withdrawal (*n* = 2) or not meeting inclusion criteria (*n* = 23). A total of 80 volunteers (55 men and 25 women) were randomly assigned to the AGM and placebo groups (Figure 1). The trial was conducted from November 2019 to May 2020. Of the 80 enrolled subjects, 4 dropped out, and 76 participants (37 in the AGM group and 39 in the placebo group) were included in the FAS analysis (Figure 1). One participant in the AGM group withdrew consent, and two participants did not meet inclusion criteria (Figure 1). No significant intergroup difference was found for compliance with intervention during the 12-week treatment period (*p* > 0.05; Table 2).

### 3.2. Baseline Characteristics of the Participants

At baseline, no significant intergroup differences were found for age, gender, height, BMI, serum glucose, serum insulin, HOMA-IR, pulse rate, systolic blood pressure (SBP), or diastolic blood pressure (DBP) (*p* > 0.05; Table 1). Although there was no statistically significant gender difference between groups, the number of male participants was fewer in the AGM group than in the placebo group. These differences affected mean body weight significantly higher in the placebo group than the AGM at baseline (*p* = 0.035; Table 1). However, BMI was not significantly different between the two groups. Therefore, the analysis was adjusted for baseline body weight. One menopausal woman was included in the AGA group. Alcohol intake and smoking status were not significantly different in the AGM and placebo groups at baseline (*p* > 0.05).

### 3.3. Nutrient Intake and BMI at Baseline and 12-Week Intervention and Compliance of Intervention

Changes in daily nutrient intake calculated from the 3-day food record (energy, carbohydrate, fat, protein, fiber, vitamin A, E, and C, zinc, and selenium) from baseline to 12-week were not significantly different between the AGM and placebo groups (Table 2). It suggested that the food intake of the participants was not different between the two groups. The 3-day food records indicated that the participants primarily had Korean-style balanced diets, including rice, soup, fermented cabbage, meats or fish, and cooked vegetables. The changes in alcohol intake, smoking status, and physical activity levels between the 12-week intervention did not differ significantly between the two study groups after the 12-week intervention (*p* > 0.05; Table 2). The changes in body weight and BMI during the 12-week intervention were not significantly different between the AGM and placebo groups (*p* > 0.05; Table 2). No significant intergroup difference was found for compliance with intervention during the 12-week treatment period (*p* > 0.05; Table 2).

### 3.4. Efficacy Evaluation of Primary Outcomes

**Figure 2:**
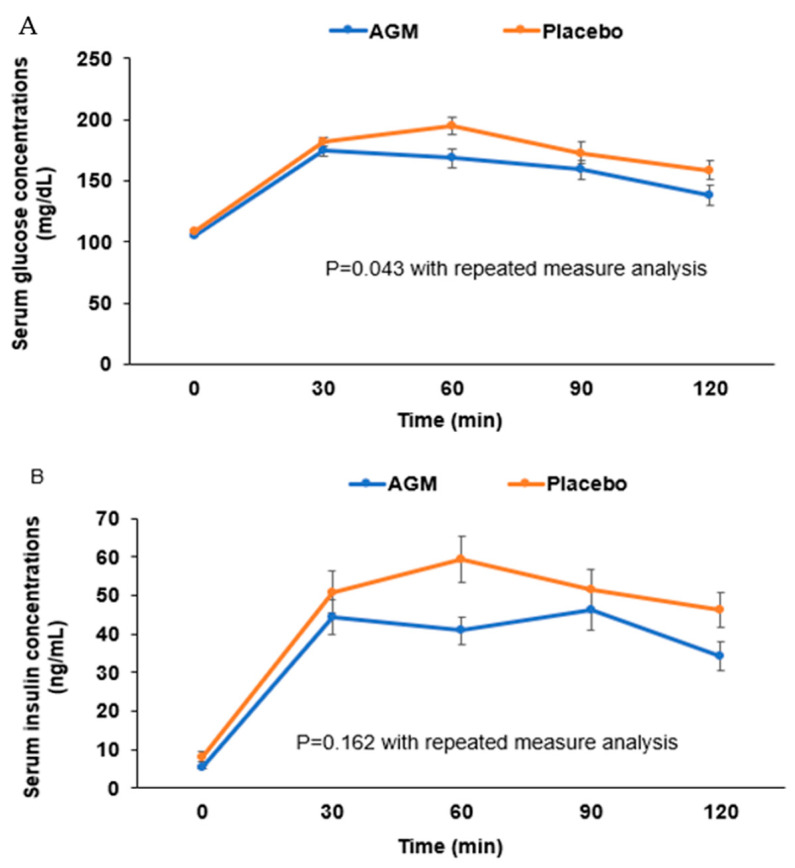
The changes of serum glucose (**A**) and insulin (**B**) concentrations during the oral glucose tolerance test after the 12-week intervention. Dots and error bars indicate means ± standard errors. Statistical significance was analyzed by linear mixed model for repeated measure data differences between groups after adjusting for baseline body weight.

Serum glucose and insulin concentrations at baseline and after the 12-week intervention period in the two groups are presented in Table 3. At the baseline of the intervention, serum glucose concentrations were not significantly in the fasting state or at 30, 60, 90, or 120 min after administering 75 g of glucose and adjusting for body weight (Table 3). However, after the 75 g glucose challenge, serum glucose concentrations at 12 weeks were quickly lower after 30 min in the AGM group while they increased until 60 min in the placebo group (Figure 2A). Serum glucose concentrations after the 12-week intervention were lower in the AGM group than the placebo group using repeated measure analysis (*p* = 0.043; Figure 2A).

Fasting serum insulin concentrations at 12-week were lower in the AGM group than at 0-week (*p* = 0.012). They were also significantly lower in the AGM group than in the placebo group after 12-week treatment by repeated measure analysis (*p* = 0.043; Table 3). After the 12-week intervention, serum insulin concentrations increased until 30 min in the AGM group, but they were elevated until 60 min in the placebo group (Table 3, Figure 2B). In repeated measure analysis of serum insulin concentration during OGTT after administering 75 g of glucose intake, they were not significantly different in the two groups (*p* = 0.162; Table 3).

The AUC of serum glucose concentrations did not significantly differ between the AGM and placebo groups after the 12-week treatment (Table 3). The AUC of serum insulin concentrations increased at 12 weeks in the AGM group versus baseline, but not significantly, and it was not significantly different between the two groups (Table 3).

After adjusting for body weight at baseline, the 12-week AUCs of serum glucose and insulin concentrations were not significantly different in the AGM and placebo groups (Table 4), and neither were blood HbA1c levels. Like fasted serum insulin concentrations, fasted serum C-peptide concentrations at 12 weeks were lower in the AGM group (−0.49 *±* 1.00, *p* = 0.005) (Table 4). However, fasted serum C-peptide concentrations at 12 weeks were not significantly different in the AGM and placebo groups (Table 4).

HOMA-IR (an insulin resistance index during a fasting state) decreased by 37.9% from baseline in the AGM at 12 weeks (*p* = 0.005), but not in the placebo group (Table 4), and HOMA-IR was significantly higher in the placebo group at 12 weeks after adjusting for body weight (*p* = 0.049). The Matsuda index is an insulin sensitivity index calculated by incorporating serum glucose and insulin concentration during OGTT and reflecting glucose deposition by the liver and skeletal muscles. A higher Matsuda index indicated better insulin sensitivity. The Matsuda index tended to be higher in the AGM group than the placebo group, but it was not significantly different (Table 4). The oral disposition index is a β-cell function index and a strong predictor of incident diabetes in Asians with low insulin secretion capacity [19]. The Disposition index increased in the AGM group after 12-week intervention (*p* = 0.043), but it did not differ in the placebo group. However, there was no significant difference between the AGM and placebo groups after 12 weeks of treatment (Table 4). HOMA-B (an insulin secretion index) was lower at 12 weeks in the AGM group than in the placebo group after adjusting for body weight (*p* = 0.044) (Table 4). The changes of HOMA-B were significantly lower in the AGM group after 12 weeks of treatment (*p* = 0.005). The lower HOMA-B and higher deposition index observed in the AGM group may have reflected a reduction in insulin resistance to prevent exhaustion of insulin supply from pancreatic β-cells to improve β-cell function in the fasted state. Serum adiponectin concentrations were unchanged at 12 weeks in both groups, and no significant intergroup difference was observed (Table 4).

### 3.5. Evaluations of Liver Damage and Inflammation

Liver damage and inflammation indices were investigated because glucose metabolism is associated with liver function. At 12 weeks, serum γ-GT, AST, and ALT concentrations and liver damage indices were significantly lower in the AGM group than at baseline (Table 5) and lower in the AGM group than in the placebo group after adjusting for body weight (Table 5), which indicated AGM ameliorated liver damage. Moreover, serum hs-CRP concentrations at 12 weeks were lower in the AGM group than in the placebo group (*p* = 0.063) after adjusting for body weight.

### 3.6. Safety and Adverse Reactions

No significant changes in safety indicators such as vital signs (blood pressure and pulse) or body weight were observed in either group (Table S1). Adverse reaction rates were similar in the two groups (*p* = 0.714) and were reported by 3 of 37 participants in the AGM group and 5 of 39 in the placebo group (Table S2). The adverse reactions encountered were abdominal discomfort, leg pain, low back pain, and catching a cold in the AGM group (one participant each) and AST, ALT, and γ-GT elevation; HbA1c and glucose increase; catching a cold or flu; gastroesophageal reflux; and triglyceride elevation in the placebo group (also in one participant each). These symptoms were not related to the AGM intake during the intervention, and they were treated with appropriate medications.

## 4. Discussion

*Aronia melanocarpa* berries and red ginseng have been reported to improve insulin resistance and promote glucose-stimulated insulin secretion [4,20,21]. The mixture of *Aronia melanocarpa* berries, red ginseng, Shiitake mushroom, and nattokinase (AGM) has also been demonstrated to have anti-diabetic activity by reducing insulin resistance and potentiating glucose-stimulated insulin secretion by improving gut microbiome dysbiosis in rats with type 2 diabetes [13]. In the present study, we examined the efficacy and safety of oral AGM on glucose metabolism and insulin resistance in prediabetic adults. AGM administration for 12 weeks did not significantly alter fasting serum glucose concentrations or OGTT results, though serum glucose concentrations determined by 60 min OGTT testing tended to be lower in the AGM group than in the placebo group. It may have been that the absence of significant changes in serum glucose concentrations was related to serum glucose concentrations within normal ranges in the participants. However, at 12 weeks, fasting serum insulin concentrations were significantly lower in the AGM group. Furthermore, at this time, serum γ-GT, ALT, and AST activities were significantly reduced, and the changed serum CPR concentrations during the 12-week intervention decreased in the AGM group (*p* = 0.069), which suggested AGM reduced insulin resistance and liver damage and thus, improved hepatic insulin sensitivity.

The risk of insulin resistance is increased by age, overweight, menopause, oxidative stress, and inflammatory status [22,23], and insulin resistance is a major causative factor of prediabetes, type 2 diabetes, and gestational diabetes [24]. Furthermore, reductions in insulin resistance can prevent the development of type 2 diabetes and cardiovascular diseases. When insulin resistance is elevated, pancreatic insulin secretion increases to normalize serum glucose levels, and if insulin secretion is insufficient, serum glucose concentrations increase and induce type 2 diabetes. Thus, functional foods that reduce insulin resistance and potentiate glucose-stimulated insulin secretion reduce the risk of type 2 diabetes and cardiovascular diseases [25]. Reportedly, Aronia normalizes serum glucose concentrations by reducing hyperglycemia-induced oxidative stress and inflammation, improving insulin sensitivity, and potentiating glucose-stimulated insulin secretion in diabetic animal models, and it has been reported to enhance hepatic insulin resistance and increase serum glucagon-like peptide-1 concentrations by reducing dipeptidyl peptidase IV activity in obese rats [26]. Red Panax ginseng has also been demonstrated to improve glucose homeostasis in type 2 diabetes, but not in prediabetic or healthy adults [21], and to suppress diabetes-related cardiovascular diseases and normalize serum glucose concentrations in diabetic patients [27]. Consistent with these previous studies, we found that 12 weeks of AGM treatment reduced insulin resistance in prediabetic participants, which suggests AGM may have anti-diabetic activity in prediabetic and healthy adults by reducing insulin resistance, despite the observation that serum glucose levels were similar in the AGM and placebo groups after intervention.

Vitamin D deficiency has also been associated with insulin resistance-related diseases, including type 2 diabetes and non-alcoholic fatty liver disease (NAFLD) in human and animal studies, but reported results are inconsistent [9,28]. Asians have lower serum 25-OH-cholecalciferol concentrations (a measure of vitamin D status) than Caucasians, and in Chinese and Indians, vitamin D deficiency is associated with insulin resistance [29,30]. However, studies about the effect of vitamin D supplementation on glucose metabolism do not produce consistent results. For example, Pramono et al. found no insulin sensitivity improvement in meta-analysis with mixed ethnicities [31] while Mirhosseini et al., in a meta-analysis, concluded vitamin D supplementation significantly enhances insulin sensitivity in Caucasians [32]. Vitamin D supplementation has the potential to improve glucose metabolism. UV-irradiated dried Shiitake mushrooms contain high levels of vitamin D2 (41 μg/g dry powder) and β-glucans (97 mg/g dry powder) [33]. Vitamin D2 in the AGM may improve glucose metabolism in the present study. However, vitamin D2 bioavailability remains controversial due to poor absorption and inactivation, although vitamin D2 is considered to have similar activity to vitamin D3 in rickets [34]. However, a meta-analysis has demonstrated that vitamin D2 intake for 12 weeks normalizes serum 25-OH-D but does not improve insulin secretion and insulin sensitivity [35]. Thus, vitamin D2 effect on glucose homeostasis needs to do further studies.

Fermented soybeans, such as chungkookjang, doenjang, and natto, contain nattokinase made from several *Bacillus* species and nattokinase fibrinolytic activity via fibrinogen and fibrin hydrolysis [36]. Shiitake mushroom also has fibrinolytic activity via prohibiting the action of platelet aggregators from delaying activated thromboplastin time, prothrombin time, and coagulation time [37]. Intake of rice bran hydrolyzed with enzymes in Shiitake mushroom improves glucose homeostasis in diabetic patients [38]. The fibrinolytic enzymes in Shiitake mushroom and nattokinase also have different fibrinolytic activity mechanisms and may promote fibrinolytic synergistically. Hyperglycemia activates platelet aggregation, and aspirin does not prohibit the activation [39]. Nattokinase and protease from Shiitake mushrooms may have a beneficial activity to suppress platelet aggregation under hyperglycemia.

β-glucan in Shiitake mushrooms and other mushrooms have been reported to reduce fat accumulation and insulin resistance and alleviate liver damage in animal models [40]. β-glucan is one of the prebiotics that decreases serum glucose concentration with increasing propionate and butyrate-producing bacteria in the gut [41]. Anthocyanins like Aronia and black currant also promote glucose metabolism by increasing the relative abundance of *Bacteroides*, *Prevotella*, and *Akkermansia* to increase butyrate production [13,42,43]. Therefore, AGM intake might promote gut microbiota composition contributing to improving glucose metabolism.

We found AGM reduced liver damage indexes, including serum γ-GT, AST, and ALT activities, suggesting it might reduce insulin resistance in healthy adults. The liver is a critical organ in the context of metabolic diseases. Obesity, type 2 diabetes, and dyslipidemia are all associated with NAFLD. This disease is difficult to diagnose by blood testing but is partly represented by liver damage indexes and serum activities of AST, ALT, and γ-GT. Normal serum ALT levels for men and women are 29~33 IU/L and 19~25 IU/L, respectively, and in the present study, levels were marginally elevated at baseline. After 12 weeks of AGM treatment, serum ALT activity reductions were much more significant in the AGM group than in the placebo group, and serum ALT activity after AGM treatment was lower than the upper level of the normal range. Liver damage is associated with hepatic insulin resistance, insulin secretion, oxidative stress, and inflammation in healthy adults. It is also related to elevated serum levels of CRP, tumor necrosis factor-α, interleukin (IL)-1β, and IL-6 [44]. Previous studies have demonstrated that Aronia and ginseng improve insulin resistance and NAFLD and reduce oxidative stress, inflammation [45], and alcohol-induced liver damage [46]. In the present study, AGM intake reduced the serum activities of γ-GT and ALT and serum CRP concentrations, which suggests AGM can reduce hepatic insulin resistance and inflammation.

Dietary intake, including high carbohydrate and low vitamin C and fiber intake, influences metabolic syndrome risk, including glucose metabolism in Koreans, although the association between high carbohydrate intake and glucose metabolism is still controversial [47,48,49]. Dietary patterns such as the Mediterranean diet have been studied for the management of metabolic diseases [50]. In dietary pattern studies, Koreans can be categorized into 3 different dietary patterns: Korean-style balanced diet, noodle/meat diet, and rice-main diet. About 50% of people have a Korean-balanced diet [51]. Kim et al. have reported that the Korean-balanced diet reduces body fat percent, BMI, and serum LDL cholesterol concentrations, but not serum glucose concentrations, compared to the typical American diet and diet recommended by the 2010 dietary guidelines for Americans in a randomized clinical trial [52]. Korean balanced diet has some beneficial effects on metabolic syndrome, cardiovascular disease, possibly, and type 2 diabetes. In the present study, food intake was estimated with 3-day food records, indicating that the participants in the AGM and placebo groups mainly had a Korean balanced diet including rice, soup, fermented cabbage and soybeans, beans, fish, cooked vegetables and fruits, and the persons who consume a Korean balanced diet belong to *Ruminococus* enterotype [53]. There was no difference in the intake of individual foods. Some foods, including fermented soybeans, beans, vegetables, and fruits, contain phytochemicals like polyphenols acting as functional foods [25,50]. They need to be determined to check their effect on metabolism. The participants were asked not to change diets and not consume any functional foods and dietary supplements in the present study. Daily intake of energy, carbohydrate, fat, fiber, and antioxidant vitamins did not differ between the two groups. These results indicated that dietary impact on glucose metabolism is considered to be minimal.

**Figure 3:**
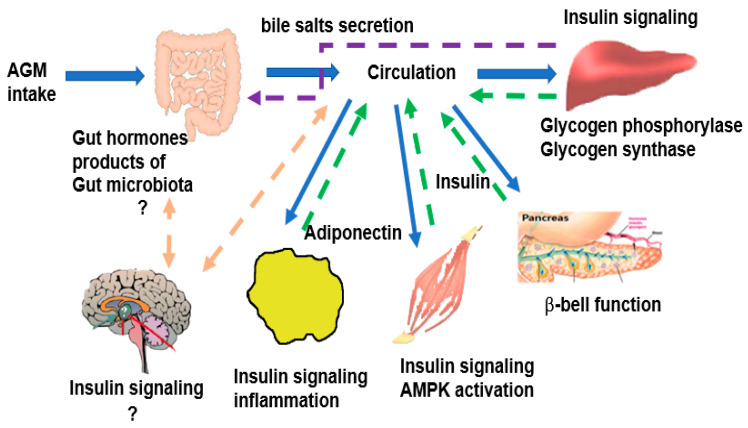
The potential mechanism of AGM in glucose homeostasis. AGM, the mixture of Aronia, red ginseng, Shiitake mushroom, and nattokinase.

The potential mechanism of AGM action was to improve insulin sensitivity in the peripheral tissues such as the liver, skeletal muscles, and adipose tissues by potentiating insulin signaling (Figure 3) [13]. After AGM intake, the AGM components may change the gut microbiota and are also absorbed in the intestines and transported to the liver through the portal vein. Finally, they are taken up by the skeletal muscles, adipose tissues, islets, and possibly, the brain from the circulation. The AGM components directly and/or indirectly improve insulin signaling in each tissue. The reversal of decreased serum adiponectin concentration suggests that adipose tissues are involved in changing insulin sensitivity in the AGM group (Figure 3). Improvement of insulin sensitivity protected against decreased β-cell function, which helped prevent type 2 diabetes. Therefore, the improvement of insulin signaling in the liver and adipose tissues may be a therapeutic target of AGM. Furthermore, AGM may influence gut microbiota composition and SCFA production by altering bile salt secretion and the gut microbiome–brain axis to modulate glucose homeostasis.

This study has several limitations. First, AGM efficacy may have been underestimated since the screening criteria required that participants were prediabetic adults with a blood HbA1c of <6.5% and fasting serum glucose concentrations within the normal range. The study shows that insulin sensitivity was promoted by lowering serum γ-GT, AST, and ALT activities, which indicated improved glucose metabolism in healthy adults with fasting serum glucose concentrations of 100 to 126 mg/dL. Second, although the study sample size requirement was calculated before the study commenced, the calculation was based on the standard deviations found in a diabetic animal study. Thus, it may have been inadequate for a study on healthy adults to identify treatment efficacy. Third, age and gender influence glucose metabolism, and they were considered to analyze the glucose metabolism between the AGM and placebo groups. Although they were not significantly different between the two groups, the number of female participants tended to be higher in the AGM group (40%) than the placebo group (22.5%) (*p* = 0.09), although the participants were allocated with randomization method. Due to the higher number of female subjects in the AGM group, mean body weight was higher in the placebo group than the AGM group, and body weight was used as a covariate for the adjustment. Fourth, the menstrual cycle was not considered in premenopausal women. However, the impact of the menstrual cycle on glucose metabolism, unlike lipid metabolism, is reported to be minimal, especially normoglycemia, but serum glucose concentrations were elevated in the luteal phase compared to the follicle phase in diabetic patients [54]. Thus, the menstrual effect on glucose metabolism might be negligible.

## 5. Conclusions

Twelve weeks of AGM intake significantly lowered insulin resistance than the placebo intake without changing fasting serum glucose concentrations versus placebo treatment in prediabetic adults. Serum activities of γ-GT, AST, ALT, and serum hs-CRP concentrations were lower in the AGM group than in the placebo group after treatment. Our results indicate AGM intake may improve insulin sensitivity, maintain β-cell function, and reduce liver damage and inflammation in prediabetic adults. A further large clinical study with long-term duration (at least 1 year) is needed to confirm AGM’s impact on glucose homeostasis by improving insulin sensitivity and β-cell function in prediabetic and type 2 diabetic patients.

## Figures and Tables

**Table 1:** General characteristics of the participants at baseline.

	AGM (*n* = 40)	Placebo (*n* = 40)	*p* Value ^(1)^
Genders (M/F)	24/16	31/9	0.091 ^(2)^
Age (years)	42.0 ± 12.7	39.2 ± 12.3	0.308
Height (cm)	166.5 ± 9.7	170.0 ± 7.7	0.076
Weight (kg)	67.6 ± 12.5	73.7 ± 13.3	0.035 *
BMI (kg/m^2^)	24.2 ± 2.71	25.4 ± 3.32	0.099
Serum glucose at fasting (mg/dL)	105.5 ± 9.20	108.6 ± 10.7	0.187
Serum insulin at fasting (μU/mL)	8.36 ± 7.83	8.13 ± 4.74	0.88
SBP (mmHg)	126.9 ± 13.2	128.2 ± 10.1	0.622
DBP (mmHg)	80.2 ± 11.2	80.58 ± 9.75	0.873
Pulse (per minute)	75.6 ± 12.0	78.03 ± 8.64	0.304
Alcohol (n, %)	18 (45)	18 (45)	1.000 ^(2)^
Alcohol (units/week)	9.58 ± 3.92	9.14 ± 5.04	0.772
Smoking (n, %)	4 (10)	10 (25)	0.078 ^(2)^
Smoking (cigarette/day)	15.0 ± 5.77	11.9 ± 5.0	0.334

Values are presented as mean ± SD or number (%). ^(1)^ Analyzed by independent t-test between groups in continuous variables. ^(2)^ Analyzed by chi-square test between groups. * *p* < 0.05. AGM, Mixture of Aronia, red ginseng, Shiitake mushroom, and nattokinase; BMI, body mass index; SBP, systolic blood pressure; DBP, diastolic blood pressure.

**Table 2:** Nutrient intake, physical activity, and body weight at baseline and 12th week.

	AGM		Placebo		
	Baseline (*n* = 37)	12-Week (*n* = 37)	Placebo (*n* = 39)	12-Week (*n* = 39)	*p* Value ^(2)^
Energy intake (kcal)	1722 ± 457 ^(1)^	1665 ± 478	1788 ± 554	1702 ± 547	0.768
Carbohydrate intake (En %)	56.9 ± 17.2	58.0 ± 17.2	56.3 ± 15.2	54.0 ± 15.2	0.607
Protein intake (En %)	17.0 ± 4.9	16.7 ± 6.2	17.5 ± 6.0	17.5 ± 6.1	0.902
Fat intake (En %)	26.1 ± 10.6	25.3 ± 9.8	29.7 ± 13.0	28.5 ± 13.0	0.917
Fiber intake (En %)	19.7 ± 7.8	18.6 ± 7.9	18.6 ± 7.5	17.7 ± 6.5	0.890
Vitamin A (ug RE)	794 ± 389	747 ± 370	759 ± 443	767 ± 354	0.553
Vitamin E (mg)	14.6 ± 6.80	15.7 ± 8.25	16.6 ± 8.58	15.9 ± 6.66	0.290
Vitamin C (mg)	99.6 ± 59.0	88.9 ± 49.4	86.5 ± 48.7	89.8 ± 42.6	0.137
Zinc (mg)	10.0 ± 2.96	9.51 ± 3.01	10.5 ± 3.47	10.1 ± 3.96	0.829
Selenium (ug)	94.1 ± 28.8	87.3 ± 30.6	97.8 ± 33.3	92.9 ± 34.0	0.791
Alcohol (units/week)	9.70 ± 4.02	6.81 ± 3.00	9.14 ± 5.04	7.03 ± 3.49	0.268
Smoking (cigarette/day)	16.7 ± 5.8	13.3 ± 7.6	11.9 ± 5.0	11.0 ± 4.4	0.563
Physical activity (min/week)	5572 ± 12725	4664 ± 7476	2874 ± 4465	4196 ± 5835	0.082
Body weight (kg)	67.8 ± 12.6	67.8 ± 12.6	74.0 ± 13.3	74.0 ± 13.6	0.945
Body mass index (kg/m^2^)	24.2 ± 2.75	24.2 ± 2.67	25.5 ± 3.31	25.5 ± 3.42	0.780
Compliance of taking supplements (%) ^(3)^	-	94.8 ± 4.33	-	96.0 ± 4.71	0.260

^(^^1)^ Values are presented as mean ± SD. ^(2)^ Analyzed by independent t-test for changed values from baseline to 12-week between the AGM and placebo groups. ^(3)^ Compliance taking test and placebo products = the actual consumed products /prescription products × 100. AGM, Mixture of Aronia, red ginseng, Shiitake mushroom, and nattokinase; En%, energy percentage.

**Table 3:** Serum glucose and insulin concentrations during oral glucose tolerance test with 75 g glucose.

Time	ARC Group (*n* = 37)				Placebo Group (*n* = 39)					
	Baseline	12-Week	Change Value	*p* Value ^(2)^	Baseline	12-Week	Change Value	*p* Value ^(2)^	*p* Value ^(3)^	Adjusted *p* Value ^(4)^
Serum glucose (mg/dL)										
0 min	105.5 ± 9.2 ^(1)^	105.1 ± 10.2	−0.37 ± 9.7	0.817	108.6 ± 10.7	108.7 ± 15.0	0.09 ± 12.8	0.964	0.859	0.859
30 min	173.5 ± 32.2	175.1 ± 29.4	1.57 ± 27.4	0.729	182.1 ± 23.7	182.1 ± 30.3	0.03 ± 26.1	0.995	0.802	0.802
60 min	172.4 ± 46.2	168.3 ± 43.9	−4.09 ± 29.4	0.402	187.1 ± 45.4	194.6 ± 45.4	7.48 ± 42.2	0.276	0.168	0.172
90 min	153.0 ± 48.1	159.2 ± 48.3	6.17 ± 30.7	0.229	168.0 ± 53.0	173.0 ± 52.2	4.97 ± 38.3	0.422	0.881	0.881
120 min	137.7 ± 49.3	138.0 ± 49.7	0.33 ± 30.8	0.949	150.7 ± 46.0	158.6 ± 49.3	7.89 ± 44.2	0.272	0.388	0.392
*p* value				<0.001 ^(^^5)^				<0.001 ^(^^5)^	0.043 ^(^^6)^	0.043 ^(^^7)^
Serum insulin (uU/mL)										
0 min	8.36 ± 7.83	5.29 ± 2.17	−3.07 ± 7.06	0.012	8.13 ± 4.74	8.18 ± 8.04	0.05 ± 6.12	0.962	0.043	0.043
30 min	44.8 ± 36.1	44.4 ± 27.9	−0.39 ± 24.9	0.925	50.0 ± 41.5	50.8 ± 33.9	0.73 ± 24.8	0.856	0.846	0.846
60 min	43.0 ± 20.0	40.9 ± 22.3	−2.11 ± 24.5	0.603	56.3 ± 35.7	59.5 ± 37.1	3.18 ± 32.9	0.55	0.431	0.431
90 min	38.3 ± 20.2	46.1 ± 31.6	7.79 ± 29.6	0.118	51.0 ± 30.2	51.4 ± 32.5	0.34 ± 30.9	0.946	0.287	0.287
120 min	37.6 ± 30.8	34.4 ± 22.8	−3.19 ± 33.2	0.563	42.1 ± 26.0	46.3 ± 27.7	4.15 ± 31.2	0.41	0.323	0.323
*p* value				<0.001 ^(^^5)^				<0.001 ^(^^5)^	0.162 ^(^^6)^	0.162 ^(^^7)^
Area under curve										
Glucose (mg*min/dL)	6011 ± 3531	6137 ± 3284	126.6 ± 2188	0.727	6991 ± 3114	7471 ± 3428	480.0 ± 2714	0.276	0.535	0.535
Insulin	3477 ± 1819	3904 ± 1842	426.3 ± 1733	0.143	4499 ± 2755	4708 ± 2911	209.2 ± 2308	0.575	0.646	0.646
(μU*min/mL)										

^(1)^ Values are presented as mean ± SD ^(2)^ Analyzed by paired t-test between baseline and 12 weeks within the group ^(3)^ Analyzed by independent t-test for the changed values from baseline to 12-week between the AGM and placebo groups between groups ^(4)^ Analyzed by ANCOVA for the changed values from baseline to 12-week between the AGM and placebo groups between groups after adjusting with the baseline of body weight ^(5)^ Analyzed by linear mixed model for repeated measure data differences within a group ^(6)^ Analyzed by linear mixed model for repeated measure data differences within a group ^(7)^ Analyzed by linear mixed model for repeated measure data differences between groups after adjusting for baseline body weight AGM, Mixture of Aronia, red ginseng, Shiitake mushroom, and nattokinase.

**Table 4:** Changes of HbA1c, C-peptide, indexes of insulin sensitivity and β-cell function, and adiponectin.

Time	ARC Group (*n* = 37)				Placebo Group (*n* = 39)					
	Baseline	12-Week	Change Value	*p* Value ^(^^2)^	Baseline	12-Week	Change Value	*p* Value ^(^^2)^	*p* Value ^(^^3)^	Adjusted *p* Value ^(4)^
HbA1c (%)	5.52 ± 0.38 ^(1)^	5.57 ± 0.38	0.05 ± 0.20	0.141	5.63 ± 0.30	5.63 ± 0.42	0.00 ± 0.29	0.971	0.373	0.377
C-peptide (ng/mL)	2.40 ± 1.10	1.90 ± 0.53	−0.49 ± 1.00	0.005	2.49 ± 1.03	2.30 ± 1.10	−0.19 ± 0.71	0.108	0.132	0.128
HOMA-IR	2.24 ± 2.31	1.39 ± 0.60	−0.85 ± 2.14	0.020	2.19 ± 1.35	2.26 ± 2.56	0.07 ± 1.92	0.812	0.049	0.049
Matsuda index	6.32 ± 3.65	6.90 ± 4.04	0.58 ± 3.28	0.289	5.12 ± 2.63	5.32 ± 3.07	0.20 ± 2.77	0.651	0.588	0.588
HOMA-B	69.2 ± 54.8	46.4 ± 19.5	−22.8 ± 46.2	0.005	66.9 ± 41.0	64.2 ± 46.1	−2.77 ± 39.0	0.661	0.044	0.044
Oral disposition index	2.76 ± 1.73	3.60 ± 2.59	0.84 ± 2.27	0.031	2.26 ± 1.25	2.70 ± 2.82	0.43 ± 2.24	0.234	0.433	0.433
Serum adiponectin (ng/mL)	55.2 ± 37.1	55.3 ± 38.1	0.13 ± 12.7	0.95	49.4 ± 44.5	45.9 ± 39.2	−3.42 ± 10.6	0.052	0.19	0.19

^(1)^ Values are presented as mean ± SD. ^(2)^ Analyzed by paired t-test between baseline and 12 weeks within the group ^(3)^ Analyzed by independent t-test for the changed values from baseline to 12-week between the AGM and placebo groups between groups ^(4)^ Analyzed by ANCOVA for the changed values from baseline to 12-week between the AGM and placebo groups between groups after adjusting with body weight at baseline. AGM, Mixture of Aronia, red ginseng, Shiitake mushroom, and nattokinase; HbA1C, hemoglobin A1c; CRP, C-reactive protein; HOMA-IR, homeostatic model assessment for insulin resistance; HOMB-B, homeostasis model assessment of β-cell function.

**Table 5:** Changes in liver damage and inflammation indices.

	AGM Group (*n* = 37)				Placebo Group (*n* = 39)				
	Baseline	12-Week	Change Value	*p* Value ^(^^2)^	Baseline	12-Week	Change Value	*p* Value ^(^^2)^	*p* Value ^(^^3)^
γ-GT (U/L)	28.8 ± 24.7 ^(^^1)^	23.9 ± 16.4	−4.90 ± 12.4	0.022 *	33.6 ± 24.7	34.4 ± 25.7	0.83 ± 14.4	0.721	0.068
AST (U/L)	26.6 ± 9.0	21.8 ± 5.2	−4.77 ± 8.96	0.003 **	24.9 ± 6.6	24.3 ± 8.0	−0.58 ± 6.12	0.556	0.021 *
ALT (U/L)	27.1 ± 13.8	20.6 ± 10.0	−6.48 ± 10.7	0.001 **	28.9 ± 15.6	29.4 ± 18.1	0.52 ± 14.6	0.824	0.020 *
hs-CRP (mg/L)	0.87 ± 0.78	0.72 ± 0.64	−0.15 ± 0.84	0.299	0.85 ± 0.99	1.34 ± 2.02	0.50 ± 1.98	0.126	0.069

^(1)^ Values are presented as mean ± SD. ^(2^^)^ Analyzed by paired t-test between baseline and 12 weeks within the group. ^(^^3)^ Analyzed by independent *t*-test for the changed values from baseline to 12-week between the AGM and placebo groups between groups. * *p* < 0.05, ** *p* < 0.01. AGM, Mixture of Aronia, red ginseng, Shiitake mushroom, and nattokinase.

## Data Availability

Data are available from the corresponding author on reasonable request with a reasonable reason.

## Supplementary Materials

The following are available online at https://www.mdpi.com/article/10.3390/foods10071558/s1, Table S1: The changes of body weight, blood pressure, laboratory profiles of the participants during the 12-week intervention, Table S2: Adverse events.
