# Supplementary material for: Efficacy and Safety of Aronia, Red Ginseng, Shiitake Mushroom, and Nattokinase Mixture on Insulin Resistance in Prediabetic Adults: A Randomized, Double-Blinded, Placebo-Controlled Trial
Source: Foods. 2021 Jul 5;10(7):1558. doi: 10.3390/foods10071558 (PMC8306342; doi:10.3390/foods10071558)
Supplement: Supplementary file 1 [file foods-10-01558-s001.zip › foods-1205182-supplementary.pdf]

Supplemental Table 1. The changes of body weight, blood pressure, laboratory profiles of the participants during the 12-week intervention

	AGM (n=37)				Placebo group (n=39)				
	Baseline	12-week	Change value	*P*-value[1)]	Baseline	12-week	Change value	*P*-value[1)]	*P*-value[2)]
Weight (kg)	67.8±12.6	67.8±12.6	0.01±2.32	0.974	74.0±13.3	74.0±13.6	-0.03±2.46	0.949	0.945
BMI (kg/m^2)	24.2±2.75	24.2±2.67	0.03±0.83	0.806	25.5±3.31	25.5±3.42	0.08±0.46	0.301	0.780
SBP (mmHg)	123.68±12.41	123.31±10.28	-0.36±13.97	0.871	123.80±9.88	126.24±10.65	2.44±12.22	0.215	0.343
DBP (mmHg)	77.58±11.11	76.63±9.25	-0.95±10.19	0.560	78.90±9.92	81.13±8.90	2.23±9.55	0.147	0.154
Pulse (per min)	79.9±12.7	78.3±10.1	-1.64±11.4	0.368	77.4±9.21	78.4±11.7	1.04±10.8	0.546	0.283
Serum total protein (g/dL)	7.46±0.41	7.11±0.36	-0.35±0.32	<.0001***	7.51±0.40	7.19±0.36	-0.32±0.30	<.0001***	0.656
Serum albumin (g/dL)	4.41±0.21	4.29±0.19	-0.11±0.18	0.0003***	4.49±0.22	4.38±0.18	-0.10±0.19	0.001**	0.809
BUN (mg/dL)	13.29±3.36	13.29±3.02	0.00±3.71	0.998	14.07±3.15	13.84±3.25	-0.23±3.58	0.683	0.777
Serum creatinine (mg/dL)	0.91±0.17	0.88±0.17	-0.03±0.11	0.131	0.92±0.12	0.90±0.14	-0.02±0.08	0.164	0.723
Serum CK (U/L)	186.85±247.17	119.03±50.72	-67.82±242.36	0.085	134.85±101.20	129.26±74.73	-5.59±88.32	0.691	0.133
Serum LD (U/L)	154.90±27.40	144.46±22.64	-10.44±31.13	0.040*	149.05±31.00	146.37±25.39	-2.68±32.14	0.601	0.276
WBC (K/UL)	6.42±1.51	5.73±1.21	-0.68±1.21	0.001**	6.75±1.33	6.04±1.35	-0.72±0.86	<.0001***	0.882

RBC (M/UL)	4.89±0.48	4.71±0.49	-0.18±0.31	0.001**	5.02±0.45	4.82±0.42	-0.19±0.29	0.0001***	0.830
Blood hemoglobin (g/dL)	14.49±1.23	13.97±1.19	-0.52±0.73	<.0001***	14.83±1.23	14.40±1.20	-0.42±0.59	<.0001***	0.529
Hematocrit (%)	44.66±3.91	42.85±4.07	-1.81±3.05	0.001**	45.71±3.91	44.02±3.63	-1.70±2.78	0.0004***	0.861
Serum platelets count (K/UL)	264.03±53.44	254.29±51.65	-9.74±27.44	0.031*	254.65±41.88	245.82±42.81	-8.83±27.25	0.047*	0.883
Urine pH	6.08±0.77	5.99±0.76	-0.09±1.08	0.606	5.96±0.84	5.72±0.59	-0.24±1.01	0.143	0.525

1) Values are presented as mean ± SD.

2) Analyzed by paired t-test between baseline and 12 weeks within the group

3) Analyzed by independent t-test for the changed values from baseline to 12-week between the AGM and placebo groups

*P<0.05, **P<0.01. AGM, Mixture of Aronia, red ginseng, shiitake mushroom, and nattokinase (Chakreis)

SBP, systolic blood pressure; DBP, diastolic blood pressure; BUN, blood urinary nitrogen; CK, creatine kinase; LD, lactate dehydrogenase; WBC, white blood cell counts; RBC, red blood cell counts.

Supplemental Table 2. Adverse events

	AGM (n=37)	Placebo(n=39)	Total (n=76)	*P*-value[1)]
Adverse events: N (%)	3 (8.10%)	5 (12.8%)	8 (10.5%)	0.714

Values are presented as number (%)

[1)] Analyzed by chi-square test between groups

AGM, Mixture of Aronia, red ginseng, shiitake mushroom, and nattokinase (Chakreis)
